# Angiogenesis and EMT regulators in the tumor microenvironment in lung cancer and immunotherapy

**DOI:** 10.3389/fimmu.2024.1509195

**Published:** 2024-12-16

**Authors:** Taotao Yan, Jiahai Shi

**Affiliations:** ^1^ Medical School of Nantong University, Nantong University, Nantong, China; ^2^ Department of Thoracic Surgery, Affiliated Hospital of Nantong University, Nantong, Jiangsu, China; ^3^ Nantong Key Laboratory of Translational Medicine in Cardiothoracic Diseases, and Research Institution of Translational Medicine in Cardiothoracic Diseases, Affiliated Hospital of Nantong University, Nantong, Jiangsu, China

**Keywords:** lung cancer, angiogenesis, EMT, immunotherapy, TME

## Abstract

Lung cancer remains the primary cause of cancer-related mortality, with factors such as postoperative tumor recurrence, metastasis, and therapeutic drug resistance exacerbating patient outcomes. Immunotherapy has emerged as a transformative approach, challenging conventional treatment paradigms for lung cancer. Consequently, advancing research in lung cancer immunotherapy is imperative. Recent studies indicate that numerous regulators within the tumor microenvironment (TME) drive tumor angiogenesis and epithelial-mesenchymal transition (EMT); these processes are interdependent, reciprocal, and collectively contribute to tumor progression. Tumor angiogenesis not only supplies adequate oxygen and nutrients for cellular proliferation but also establishes pathways facilitating tumor metastasis and creating hypoxic regions that foster drug resistance. Concurrently, EMT enhances metastatic potential and reinforces drug-resistance genes within tumor cells, creating a reciprocal relationship with angiogenesis. This interplay ultimately results in tumor invasion, metastasis, and therapeutic resistance. This paper reviews key regulators of angiogenesis and EMT, examining their impact on lung cancer immunotherapy and progression, and investigates whether newly identified regulators could influence lung cancer treatment, thus offering valuable insights for developing future therapeutic strategies.

## 1 Background

Lung cancer ranks as the second most prevalent cancer globally, leading in incidence among men and following breast cancer among women (1). Non-small cell lung cancer (NSCLC), the predominant form, is a highly malignant subtype with a poor prognosis, encompassing adenocarcinoma, large cell carcinoma, and squamous cell carcinoma, which together constitute approximately 80%-85% of all lung cancers (2, 3). While early-stage cancers are often managed effectively with surgical resection or radiotherapy, advanced cancers can only be treated by chemotherapy (4), which becomes progressively less effective as resistance develops and the disease worsens (5). Recent advancements in cancer immunotherapy, particularly immune checkpoint inhibitors (ICIs), have introduced promising alternatives for lung cancer management. ICIs, effective in treating unresectable advanced lung cancer as well as in perioperative settings, target PD-1 or PD-L1 pathways (6) to mitigate treatment side effects and improve survival rates. Nevertheless, achieving a definitive cure for lung cancer remains a distant goal (7). Research into molecular mechanisms underlying lung cancer progression is essential for identifying potential therapeutic targets, offering significant insights for future treatment modalities.

The tumor microenvironment (TME) plays a pivotal role in tumor cell proliferation and invasion, constituting a complex system composed of cellular components such as tumor, immune, and stromal cells, and non-cellular elements including tumor-associated fibroblasts, adjacent mesenchymal tissues, vascular networks, and various chemokines (8, 9). Within the TME, immune cells contribute to cancer progression through mechanisms that support tumor proliferation and metastasis, such as immune evasion, epithelial-mesenchymal transition (EMT), angiogenesis, and immunosuppression (10). In recent years, strategies targeting TME regulation have gained significant interest in cancer immunotherapy. Despite the initial efficacy of immunotherapeutic agents, therapeutic success is frequently compromised by emerging drug resistance within the host (11). Continued research on angiogenesis and EMT regulatory mechanisms offers potential pathways to address these challenges, advancing the effectiveness of lung cancer therapies.

The development of the human vascular system is an intricately orchestrated process, necessitating precise temporal and spatial coordination among various cell types to form functional blood vessels. Angiogenesis, the formation of new vasculature from pre-existing vessels, is fundamental to both physiological and pathological processes, such as wound healing, organ development, ischemic conditions, inflammatory diseases, fibrosis, and cancer (12). This multistep process initiates new capillary growth from multifunctional pre-existing vessels, which significantly contributes to tumor recurrence and metastasis (13). Tumor expansion demands substantial nutrients and oxygen, necessitating an adequate blood supply within the TME. This supply is facilitated through angiogenesis, where the recruitment of new vessels from existing ones provides tumors with essential resources, a process driven by a complex signaling network of growth factors (14). Recently, anti-angiogenesis has emerged as a promising immunotherapeutic strategy, aiming to normalize abnormal vasculature, inhibit tumor growth and metastasis, and restrict tumor blood supply through anti-angiogenic agents (15). This approach is now applied in treating various solid tumor types (16).

EMT is a form of cellular reprogramming that allows epithelial cells to acquire a mesenchymal phenotype, essential for embryonic development and adult tissue maintenance. This process triggers cytoskeletal remodeling and mitochondrial division to meet the high energy demands of EMT, fueling further transition. EMT plays a pivotal role in tumor progression, endowing cancer cells with enhanced invasiveness and relative drug resistance (17, 18).

Emerging experimental data increasingly demonstrate that the interaction between angiogenesis and EMT in tumors significantly enhances tumor invasion, metastasis, and drug resistance. For instance, hypoxic conditions stimulate EMT through hypoxia-inducible factors (HIFs), which mediate diverse signaling pathways pivotal to angiogenesis (19). Hypoxia or HIF overexpression alone can induce EMT and promote invasiveness across various cell types. The HIF pathway indirectly drives EMT *via* multiple cellular signaling pathways, including Notch, TGF-β, integrin-linked kinases, tyrosine kinase receptors, Wnt, and Hedgehog (20). In hypoxic environments, HIF-1α upregulates anti-apoptotic genes and activates PD-L1 in tumor cells, enabling immune evasion and enhancing invasion and migration (21). Angiogenesis is frequently accompanied by an inflammatory response, with pro-inflammatory chemokines like IL-8 prompting EMT in tumor cells (22). This review consolidates current research on key regulatory factors in lung cancer, examining various pathways that drive tumor angiogenesis and EMT, ultimately contributing to tumor growth, metastasis, drug resistance, and advancements in immunotherapy.

## 2 Angiogenesis-related regulatory factors

**Figure 1:**
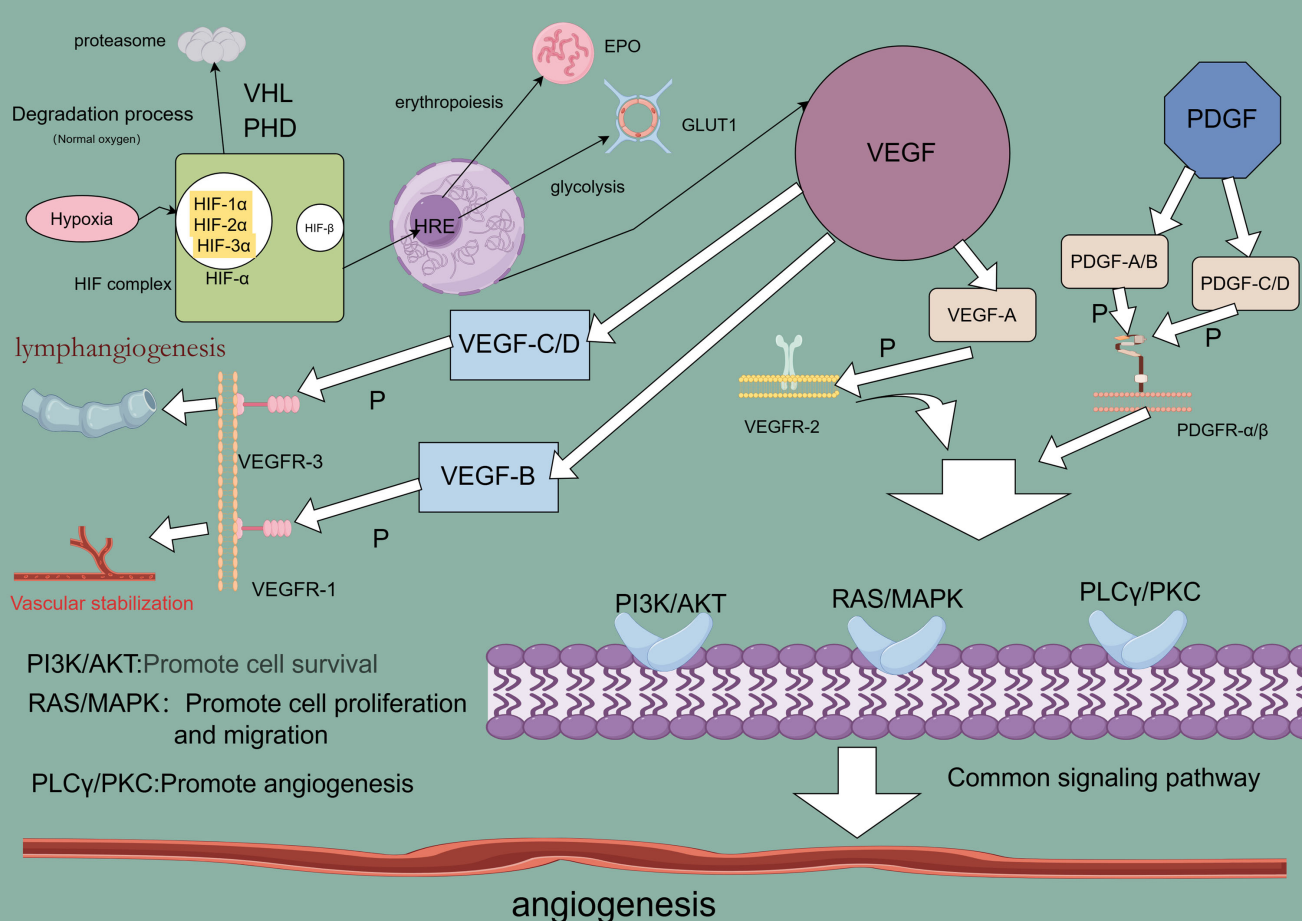
Angiogenesis-related regulatory factor signaling pathway.

Angiogenesis, the formation of new capillaries from preexisting blood vessels, is crucial for the growth and metastasis of many solid tumors. Tumor-derived angiogenic factors drive endothelial cell migration and proliferation, establishing new capillaries that support tumor expansion, invasion, and metastasis. This process initiates when pro-angiogenic molecules outweigh anti-angiogenic counterparts (23, 24). Key angiogenic regulators, such as vascular endothelial growth factor (VEGF), platelet-derived growth factor (PDGF), fibroblast growth factor (FGF), HIF, and angiopoietin, are vital in hypoxia-induced angiogenesis (**Table 1**) (25). Under hypoxic conditions, signaling pathways activate relevant receptors, and HIF directly promotes transcription of angiogenesis-related genes (e.g., VEGF), upregulating these regulators to advance angiogenesis and mitigate tissue hypoxia (26). Investigating these regulatory and signaling pathways may provide insights for enhancing therapeutic drug development for lung cancer (**Figure 1**).

**Table 1:** Regulators of angiogenesis.

Regulatory factor	Family	Signaling pathway	Type of Cancer	Targeted drug
**VEGF**	VEGF-A VEGF-B VEGF-C VEGF-D PlGF	MAPK/ERK、PI3K/AKT、PKC/FAK	NSCLC, colorectal cancer, breast cancer, renal cell carcinoma, gastric cancer, hepatocellular carcinoma, ovarian cancer, pancreatic cancer	Monoclonal antibodies (mAb) and tyrosine kinase inhibitors (TKI) such as bevacizumab, ramucirumab, and axitinib
**PDGF**	PDGF-A PDGF-B PDGF-C PDGF-D	PI3K/AKT、Ras/MAPK、PLC-γ/DAG/PKC and JAK/STAT	NSCLC, GIST, pancreatic, breast, ovarian, hepatocellular and neuroendocrine tumors	Anrotinib, imatinib, sunitinib, pazopanib, sorafenib
**HIF**	HIF-1 HIF-2 HIF-3	HIF-VEGF, HIF-glycolysis, HIF-cell survival, HIF-EMT, HIF-erythropoiesis, HIF-oxidative stress	NSCLC, RCC, HCC, Breast Cancer, Prostate Cancer, Gastric Cancer, Colorectal Cancer, Ovarian Cancer	HIF inhibitor: bedaquiline. VEGF inhibitor: bevacizumab.
**FGF**	FGF-1, FGF-4, FGF-7, FGF-8, FGF-9, FGF-19, FGF11-14	Ras/MAPK PI3K/AKT PLC-γ JAK/STAT	NSCLC, HCC, Bladder Cancer, Bile Duct Cancer, Breast Cancer, Prostate Cancer, Stomach Cancer	Erdatinib, Panitinib, Romatinib, Inritinib
**ANGPT**	ANGPT-1 ANGPT-2 ANGPT-3 ANGPT-4	PI3K/AKT MAPK/ERK Rhoa	NSCLC, Breast Cancer, Pancreatic Cancer, Colorectal Cancer, RCC, Stomach Cancer, Ovarian Cancer, Melanoma	Trebananib, CVX-060, MEDI3617, REGN910, BAY75762

### 2.1 Vascular endothelial growth factor

Angiogenesis is a critical driver of lung cancer cell growth, invasion, and metastasis, with VEGF serving as a key mediator in this process. In NSCLC, elevated levels of angiogenic markers, including VEGF, correlate with poorer prognoses (27), underscoring VEGF’s central role in tumor neovascularization (28). The VEGF family, comprising VEGF-A, VEGF-B, VEGF-C, VEGF-D, and placental growth factor (PlGF), exhibits specific functions within various tissues. VEGF-A, the most potent angiogenic factor, is essential for regulating endothelial cell sprouting, mitogenesis, migration, vasodilation, and vascular permeability (29, 30). In the heart, VEGF-B supports neuronal survival and cardiovascular growth through angiogenesis. It regulates cardiac angiogenesis and sympathetic innervation by inducing tissue-specific angiogenic responses in the myocardium, upregulating nerve ciliary protein (Nrp-1) expression, and mediating VEGFR-1 and Nrp-1 to promote nerve growth and protection. This mechanism contributes to alleviating ischemic heart disease (31), thereby aiding ischemic heart disease recovery (32). VEGF-C and VEGF-D facilitate tumor growth and metastasis through VEGFR-3-mediated lymphangiogenesis and lymphatic metastasis (33). Upon binding to its receptor, VEGF activates downstream signaling pathways such as MAPK/ERK, PI3K/AKT, PKC, and FAK, which collectively support angiogenesis. Although VEGF/VEGFR is not the sole regulatory axis, it plays an indispensable role in angiogenic signaling (34). Consequently, antivascular drugs targeting VEGF or VEGFR have emerged as promising therapeutic options, primarily including monoclonal antibodies (mAb) and tyrosine kinase inhibitors (TKIs), such as bevacizumab, ramucirumab, and acitretinib. Monoclonal antibodies exhibit high specificity by binding directly to VEGF or VEGFR, preventing receptor interaction and thereby exerting anti-angiogenic effects through receptor blockade. Tyrosine kinase inhibitors, conversely, bind to receptor tyrosine kinases, inhibiting their kinase activity and thus impeding downstream signaling (35, 36). Research confirms that VEGF and VEGFR are key regulators in lung cancer angiogenesis, serving as primary therapeutic targets for antivascular drug development to inhibit tumor growth, metastasis, and drug resistance. However, tumors often develop multiple drug resistance (MDR) through mechanisms such as decreased intracellular drug concentrations, altered drug targets, and imbalances in metabolic detoxification and DNA repair. Overexpression of transporter proteins in tumor cells further limits drug efficacy by blocking drug entry and actively expelling intracellular drugs. These transporters expel lipophilic chemotherapeutic agents, reducing their effective intracellular concentrations and establishing resistance (37). To counteract drug resistance, current treatments employ sequential maximal dose-density regimens to maximize cell destruction and minimize resistance onset (38). Dual targeting of the VEGF and ANG2 pathways has proven more effective than single-target approaches, enhancing antiangiogenic therapy outcomes (39). Despite progress, many molecular mechanisms underlying VEGF-targeted antivascular therapies remain to be elucidated, necessitating further investigation (40).

### 2.2 Platelet-derived growth factor

PDGF, a fundamental protein stored in platelet α-granules (41), along with its receptors (PDGFRα and PDGFRβ), is expressed in numerous malignant cells and tissues, including NSCLC, gastrointestinal stromal tumors (GIST), and pancreatic, breast, ovarian, hepatocellular, and neuroendocrine cancers (42). In NSCLC, overexpression of PDGFRα/β and PDGF-A/B correlates with poor prognosis; PDGF-AA, for instance, regulates VEGF expression *via* autocrine signaling, advancing the transformation of precancerous lesions into aggressive malignancies (43). Additionally, mutations in the PDGFR-α gene enhance PDGFRα expression, triggering ligand-independent PDGF signaling that fosters tumor growth in NSCLC (44). In GIST, PDGF ligand binding to PDGFRα and PDGFRβ activates the STAT pathway, influencing disease progression (45). In breast cancer, high PDGF-C expression correlates with lymph node metastasis, HER2 amplification, and elevated Ki-67 proliferation, driving progression *via* autocrine and paracrine signaling (46). In cholangiocarcinoma, hypoxia-induced PDGF-D upregulation in cholangiocarcinoma (CAA) cells activates a paracrine loop in the tumor-associated stroma, coordinating lymphangiogenesis and accelerating regional lymph node metastasis (47). PDGF activates signaling through PDGFR-mediated cellular pathways, where receptor binding initiates dimerization and phosphorylation, creating sites for downstream signaling molecule attachment and activating pathways such as PI3K/AKT, Ras/MAPK, PLC-γ, DAG, PKC, and JAK/STAT (48). Studies reveal that inhibiting the PDGF/PDGFR pathway effectively hinders tumor cell proliferation and angiogenesis (49). TKIs, which neutralize PDGFR antibodies and antagonize PDGFR kinase activity (50), have shown promise in targeted therapies, notably improving the outcomes of patients with NSCLC. Anlotinib, for example, is utilized as a third-line treatment for advanced NSCLC, targeting VEGFR to inhibit angiogenesis and lung cancer cell proliferation, demonstrating efficacy in advanced cases (51, 52). However, TKI-related toxicity and resistance present significant challenges in prolonged therapy, necessitating further in-depth research to optimize efficacy and mitigate these issues (53).

### 2.3 Hypoxia-inducible factor

Hypoxia is often indicative of solid tumor presence, activating the HIF family to modulate gene expression in both tumor cells and immune cells within the TME, thereby influencing tumor progression and therapeutic response (54). The HIF family consists of isoforms HIF-1, HIF-2, and HIF-3, each with distinct functions and transcriptional activities (55). Among these, HIF-1 has been widely identified across various cancers and plays a pivotal role in cancer development, acting as a key transcription factor (TF) that drives cancer progression and serves as a target for therapeutic intervention. HIF-1 promotes cancer cell growth, survival, angiogenesis, metastasis, and treatment resistance (56). In the hypoxic TME, rapid tumor cell proliferation outpaces the oxygen supply from surrounding blood vessels, creating an imbalance that triggers a cellular adaptive response coordinated by HIF-1 (57). HIF-1 itself is a heterodimer of α and β subunits, with HIF-1α particularly induced under hypoxia to regulate genes related to cancer cell proliferation and angiogenesis (58). HIF-1α transcriptionally activates several pro-angiogenic molecules by binding directly to promoter regions. Specifically, HIF-1α binds to the hypoxia response element (HRE) on VEGF and VEGFR1 gene promoters, inducing VEGFA and VEGFR1 expression, which promotes tumor angiogenesis through VEGF and ANGPTL4 (59). Under hypoxic conditions, HIF-1α stabilizes and initiates the expression of multiple genes through a gene expression cascade involving the MAPK pathway and VEGF signaling (60). In this process, HIF-1α binds to the hypoxia-responsive element (HRE) within the VEGF promoter, forming an HIF-1α/HRE complex that directly upregulates VEGFR-1 expression in tumor cells. This amplification of VEGF signaling promotes both solid tumor angiogenesis and pathological angiogenesis (61). Ropivacaine, a local anesthetic, has been shown to inhibit HIF-1α signaling in lung cancer cells, along with downstream VEGF signaling, thus reducing angiogenesis in malignant lung cancers (62). Additionally, HIF-1α activation of the Hippo-YAP pathway accelerates malignant progression in NSCLC, while silencing HIF-1α induces ferroptosis and inhibits NSCLC invasion (63). These findings underscore the extensive interplay of HIF-1 signaling pathways in lung cancer development, suggesting that targeting HIF-1 could open new avenues for the development of effective HIF inhibitors and therapeutic strategies (64).

### 2.4 Other angiogenesis regulators

Among the various regulators of angiogenesis, basic fibroblast growth factor (FGF2) is considered the first identified pro-angiogenic molecule, promoting angiogenesis by activating FGF receptor 1 (FGFR1) signaling in endothelial cells (65). To counteract the effects of the highly expressed FGF2/FGFR1 pathway, research indicates that VEGF-B can act as a unique angiogenic factor; although it typically has limited angiogenic activity, it can inhibit tumor growth and angiogenesis under specific conditions by suppressing FGF2-induced Erk phosphorylation and thus reducing FGF2-driven angiogenesis (66, 67). This mechanism offers a potential therapeutic strategy for controlling excessive angiogenesis, contributing to targeted therapies aimed at preventing lung cancer cell metastasis and dissemination. In the context of advancing lung cancer immunotherapy research, miRNAs, a subset of non-coding RNAs, have emerged as key regulators of cancer cell growth, metastasis, angiogenesis, and apoptosis. For instance, miR-937-3p, often highly expressed in patients with lung adenocarcinoma (LUAD), activates the PI3K/AKT pathway by targeting the downstream gene SOX11, thereby enhancing NSCLC angiogenesis and facilitating metastasis (68, 69). Additionally, dietary compounds such as flavonoids, retinoids, triterpenoids, omega fatty acids, and carotenoids have demonstrated promising roles in anti-angiogenic therapy within current cancer immunotherapy approaches (70).

## 3 EMT-related regulators

EMT is a process in which epithelial cells lose their connectivity and polarity while gaining mesenchymal characteristics and invasive potential. EMT progresses through distinct states—fully epithelial, partial EMT, partial MET, and fully mesenchymal (full EMT)—each with unique functional traits, plasticity, and heterogeneity that contribute to cancer invasion, recurrence, and drug resistance (71, 72). EMT is generally mediated by multiple signaling pathways, including the TGF-β, bone morphogenetic protein (BMP), receptor tyrosine kinase (RTK), STAT3, extracellular matrix (ECM)-mediated, and hypoxia signaling pathways. These pathways regulate TFs, influencing gene expression to increase EMT-related markers (73). Consequently, targeting EMT presents a promising therapeutic strategy, potentially offering improved recovery opportunities for patients with cancer.

### 3.1 Transforming growth factor β

**Figure 2:**
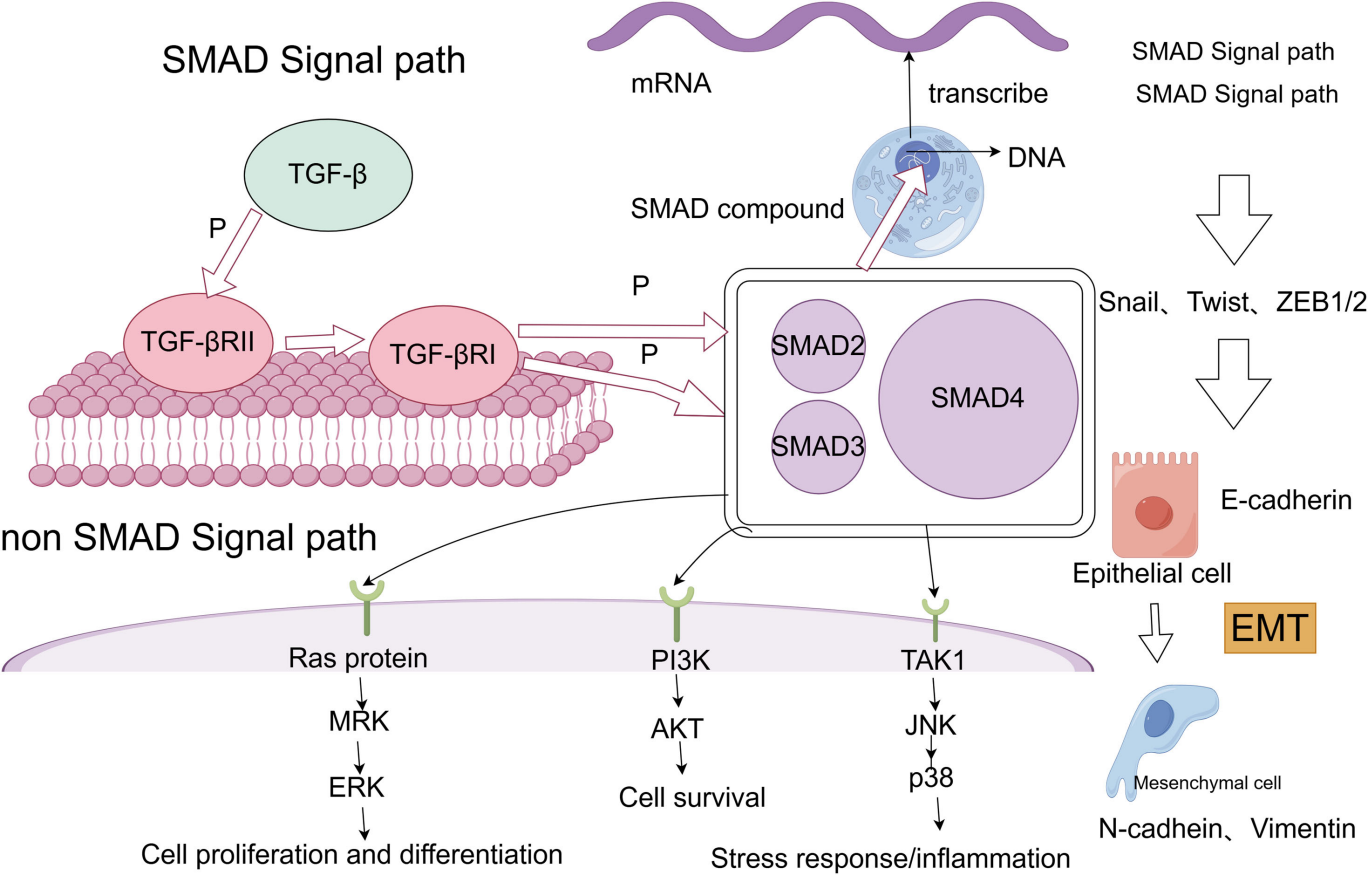
SMAD and non-SMAD pathways of TGF-β lead to EMT process.

TGF-β is widely recognized as a central driver of cancer cell plasticity through EMT. The TGF-β family includes 33 evolutionarily conserved proteins, such as TGF-β1, TGF-β2, TGF-β3, activins, bone morphogenetic proteins (BMPs), inhibins, growth and differentiation factors (GDFs), and mullerian inhibitory substances (MISs) (74). TGF-β, particularly prominent in advanced cancers, is closely linked to metastasis and chemotherapy resistance. During EMT, TGF-β activates the SMAD pathway following ligand-induced receptor activation, where SMAD proteins transmit signals to the nucleus to regulate target gene expression (75). TGF-β signaling begins with the activation of membrane-bound type I (TGFβRI) and type II (TGFβRII) receptors, leading to SMAD2 and SMAD3 activation. These form a complex with SMAD4, translocating to the nucleus to interact with DNA-binding TFs and co-regulators, modulating gene expression (76). Additionally, the TGF-β receptor complex activates non-SMAD pathways, including RAS/MAPK, TAK1/JNK/p38MAPK/IKK, and PI3K/Akt (77) (**Figure 2**). Given its overexpression and pro-tumorigenic effects across many tumor types, TGF-β is a promising therapeutic target. Combining TGF-β inhibitors with immune checkpoint blockade or chemotherapy can effectively reduce cancer cell plasticity (78). Neferine, a bisbenzylisoquinoline alkaloid, has been found to downregulate TGF-β in NSCLC, modulating MST1 to induce ROS formation, thereby promoting apoptosis and preventing proliferation, metastasis, and EMT (79). Additionally, TGFβ1-induced upregulation of PD-L1 in tumor cells has emerged as a novel mechanism of immunosuppression in NSCLC. Bintrafusp alfa (M7824), targeting both PD-L1 and TGF-β, has shown efficacy in inhibiting tumor mesenchymalization, reducing PD-L1-dependent immunosuppression, and overcoming chemoresistance in NSCLC (80). These studies, along with the development of more potent and specific TGF-β inhibitors, hold potential for treating tumors that thrive in TGF-β-rich environments (81).

### 3.2 EMT-related transcription factors

EMT-TFs (**Table 2**) include the zinc finger proteins SNAI1 (Snail1) and SNAI2 (Snail2, or Slug), twist-related proteins 1 and 2 (Twist1/2), and the zinc finger e-box binding homology cassette 1/2 (ZEB1/2). Upregulation of these EMT-TFs drives cancer cells to transition from a differentiated epithelial state to a stem cell-like state, enhancing metastatic potential (82). Among them, Snail1 plays a critical role, with its expression preceding other EMT-TFs. Snail1 activates additional EMT-TFs and suppresses epithelial genes like epithelial cadherin (E-cadherin), allowing tumors to adopt a mesenchymal morphology and invasive capacity (83). Snail1 also recruits chromatin-modifying enzymes to the E-cadherin promoter, promoting DNA methylation and transcriptional repression of E-cadherin, thereby driving EMT and promoting dedifferentiation of cancer cells into cancer stem cell-like (CSC) phenotypes. Snail1 expression is linked to increased invasion and metastasis across various cancer types, including lung cancer. Notably, reducing Snail1 expression has been shown to enhance the efficacy of numerous chemotherapies and immunotherapies. Although direct chemical inhibitors targeting Snail1 are scarce, inhibitors targeting Snail1-induced EMT have demonstrated promising results (84, 85). For instance, Entestat (ENT) can reverse Snail1-induced EMT, leading to increased E-cadherin expression and decreased levels of Twist, Snail, and other EMT-TFs, thereby reducing metastatic potential (86). ZEB1 acts as a key regulator, functioning as both an activator and repressor of target genes, depending on its interaction with the CDH1 promoter, miR-190 promoter, and TGF-β signaling intermediates like Smad, p300, and P/CAF (87). Twist1 is recognized as a key regulator of oncogenesis and metastasis. During EMT, Twist1 promotes the expression of EMT-related genes, such as type I collagen and N-cadherin, by directly binding to their promoters (88), enabling epithelial cells to transition to a mesenchymal phenotype. Twist1 is also crucial in regulating intercellular adhesion by influencing downstream targets like E-cadherin. Twist1 promotes EMT by inhibiting E-cadherin expression through Snail1 activity (89). In smokers, exposure to the nicotine-derived carcinogen nitrosamine ketone (NNK) upregulates Twist mRNA and protein expression, which correlates with increased migration and invasion of lung cancer cells. This underscores Twist’s role in regulating NNK-induced changes in EMT marker expression in lung cancer (90).

**Table 2:** EMT-related transcription factors and their functions, targets, and roles played in cancer.

EMT-TF	Functionality	Target	Role in Cancer
**SNAIL1 (Snail)**	Inhibition of E-cadherin expression initiates EMT	E-cadherin	Promote cancer cell invasion and metastasis (e.g. breast cancer, lung cancer)
**SNAIL2 (Slug)**	Inhibits epithelial phenotypes and promotes mesenchymal phenotypes,	E-cadherin	Correlates with drug resistance, migration ability of cancer cells
**TWIST1**	Regulates EMT and promotes N-cadherin expression	E-cadherin、N-cadherin,	Promote tumor cell migration, invasion (breast cancer, prostate cancer)
**TWIST2**	functionally similar to TWIST1, regulates EMT	E-cadherin	Promote tumor cell metastasis and invasion
**ZEB1**	Inhibition of epithelial cell markers and activation of mesenchymal markers	E-cadherin	Promoting EMT and invasion in multiple cancers
**ZEB2 (SIP1)**	Inhibits E-cadherin and promotes EMT	E-cadherin	Enhancement of cancer cell invasiveness and migration

### 3.3 New EMT regulators

With expanding research on EMT, numerous TFs have emerged as novel EMT regulators, such as specificity protein 1 (SP1) and E2F1. Recent studies highlight E2F1 as a pivotal TF for cell cycle progression in cancer, closely linked to metastasis. In NSCLC tissues and cell lines, E2F1 is notably upregulated and controls ZEB2 expression *via* an E2F1 binding site on the ZEB2 promoter, ultimately driving EMT and enhancing tumor invasion and metastasis (91, 92). SP1, part of a TF family that includes Sp2, Sp3, and Sp4, is critical for various biological functions such as cell growth, differentiation, apoptosis, and carcinogenesis, activating numerous cellular genes (93). In lung adenocarcinoma (LADC), aberrant SP1 expression induces EMT (94). Specifically, SP1-activated SGPP2 promotes LADC cell proliferation and invasion while inhibiting apoptosis (95). SP1 also functions as a direct target of miR-145-5p in NSCLC, where its overexpression decreases drug sensitivity, promotes EMT, and heightens drug resistance in cancer cells (96). As more EMT-related markers are identified, these insights pave the way for improved therapeutic protocols for cancer treatment.

## 4 Conclusion

This study investigated the pivotal roles of angiogenesis and EMT regulators within the tumor microenvironment in lung cancer progression. Key angiogenic regulators, including VEGF, PDGF, and HIF, significantly contribute to promoting angiogenesis, tumor growth, and metastasis in lung cancer. Concurrently, EMT regulators such as TGF-β, Snail, and Twist intensify cancer progression by enhancing the invasive and drug-resistant characteristics of tumor cells. Together, these processes synergize to drive tumor malignancy and facilitate immune evasion.

These findings lay a theoretical foundation for the potential application of combined anti-angiogenic and EMT-targeted therapies, particularly within immunotherapy. Targeting both angiogenesis and EMT modulators may enable future therapeutic strategies to not only suppress tumor growth and metastasis but also improve responsiveness to conventional treatments, offering more effective options for patients with lung cancer.
